# Impacts of the creation, expansion and management of English wetlands on mosquito presence and abundance – developing strategies for future disease mitigation

**DOI:** 10.1186/s13071-015-0751-3

**Published:** 2015-03-03

**Authors:** Jolyon M Medlock, Alexander GC Vaux

**Affiliations:** Medical Entomology group, Public Health England, Porton Down, Salisbury, Wiltshire SP4 0JG UK

**Keywords:** Mosquito, Wetland, UK, Ecology, Climate change, Arbovirus, *Aedes*

## Abstract

The incidence of mosquito-borne diseases is increasing in Europe, partly due to the incursion of a number of invasive species known to be vectors of dengue and chikungunya viruses, but also due to the involvement of native species in the transmission of West Nile virus and malaria. For some of these pathogens, there is a risk of the re-emergence of vector-borne diseases that were once widespread in Europe, but declined partly due to large-scale land-drainage projects. Some mosquito species exploit container habitats as breeding sites in urban areas; an adaptation to human-made micro-habitats resulting from increased urbanisation. However, many species thrive in natural wetland ecosystems. Owing to the impacts of climate change there is an urgent need for environmental adaptation, such as the creation of new wetlands to mitigate coastal and inland flooding. In some cases, these initiatives can be coupled with environmental change strategies to protect a range of endangered flora and fauna species by enhancing and extending wetland landscapes, which may by driven by European legislation, particularly in urban areas. This paper reviews field studies conducted in England to assess the impact of newly created wetlands on mosquito colonisation in a) coastal, b) urban and c) arable reversion habitats. It also considers the impact of wetland management on mosquito populations and explores the implications of various water and vegetation management options on the range of British mosquito species. Understanding the impact of wetland creation and management strategies on mosquito prevalence and the potential risk of increasing the levels of nuisance or disease vector species will be crucial in informing health and well-being risk assessments, guiding targeted control, and anticipating the social effects of extreme weather and climate change. Although new wetlands will certainly extend aquatic habitats for mosquitoes, not all species will become a major nuisance or a vector concern as a result. Understanding how the design and management of wetlands might exacerbate mosquito densities is crucial if we are to manage nuisance mosquitoes and control vector species in the event of a disease outbreak. This entomological evidence-base will ensure that control strategies achieve optimal efficacy at minimal cost.

## Introduction

Aquatic insects such as mosquitoes are acutely responsive to changes in temperature and rainfall. The rate of development of all insects is directly proportionate to temperature, and in the case of mosquitoes this governs the rate of immature development, blood digestion, and egg production, as well as incidental issues such as the rate of pathogen development within the mosquito. However, as an obligate aquatic insect, the degree of water availability has profound implications for the survival and abundance of immature mosquitoes. This is exemplified by the fact that the melting of winter snows in the Arctic can lead to the highest global abundances of mosquitoes, despite the cooler spring temperatures. In addition to the availability of water, the permanent/transient nature of water bodies impacts on the mosquito’s competitors and predators and hence any weather-driven or human-driven processes that affect the presence and distribution of aquatic habitats (including heavy rainfall or wetland management) will impact, to varying degrees, mosquito diversity and density [1,2].

Excessive rainfall and subsequent flooding have significant effects on the available mosquito habitat. However, this is not always a positive impact on mosquito density, as excessive flooding can denude aquatic habitats of mosquitoes through flushing [3,4]. Similarly, artificial storage of rain or river water in wetlands, coastal flooding and sea incursions following high spring tides may also create mosquito habitats, independent of rainfall [5]. Similarly, the irrigation of arable systems in semi-arid regions can artificially create mosquito habitats irrespective of weather events [6,7].

The effects of these processes on mosquito abundance and diversity can be demonstrated by various field-based studies on mosquitoes in England. Firstly, however, it is important to understand the drivers of flooding and the strategies employed during wetland and floodwater management.

### Drivers for wetland creation and expansion

One of the main challenges to adapting to climate change has been the development of national and regional strategies to mitigate coastal and inland flooding through the provision of new wetlands [8,9]. In addition, in an attempt to increase the amount of suitable habitat available for wildlife to cope with changes in climate (and extreme weather events), new wetlands are being created and failing wetlands are being restored through careful wetland management. In England, for example, Wetland Vision, sponsored by the UK Department of Environment, Food and Rural Affairs, Natural England, the Environment Agency and wildlife groups, such as the county-based Wildlife Trusts, have developed a clear vision to restore existing wetlands, double the number of ponds, and increase the area of coastal and grazing marsh by 2050 [9]. This mimics a number of similar initiatives across Europe.

Many of these strategies involve recreating wetland landscapes in areas where wetlands had previously dominated. For example, plans to create new coastal habitats in eastern and southern England aim to mitigate rises in sea levels, or offset loss of European protected saltmarsh and mudflat habitat in areas where coastal wetlands have been drained for agriculture [10]. Inland, in urban areas, new wetlands are being created as part of ecological mitigation for new housing developments, with a strong driver to provide mitigation habitat for European and UK protected species such as the Great Crested Newt and the Water Vole [11].

Some of the largest landscape-scale plans for wetland creation include projects such as the Great Fen project [12] and the Wicken Fen vision [13] in Cambridgeshire. The intention is to extend the existing highly biodiverse wetlands to increase the wildlife value of existing nature reserves and minimise their fragmentation, thereby restoring a lost wetland landscape, as well as increasing the available habitat for birds, rare plants and invertebrates. However, they also act as a storage facility for floodwater, a common feature of the inland and coastal wetland creation schemes. Similar examples can be found across the UK, including on post-industrial sites, such as mining flashes and gravel pit extractions, as well as across parts of Europe, particularly in areas associated with river valleys prone to flooding.

In addition to the wildlife benefit, wetlands are also valuable habitats because they provide important social, economic, and ecological services, such as flood control, water quality improvement, carbon sequestration and pollutant removal [14]. Wetlands also have high aesthetic and recreational value, which makes wetland landscapes highly desirable for improving human well-being and in turn promote local rural economies through increasing tourism and development of new settlements.

### Changing mosquito-borne disease risk in Europe

The main drawback associated with wetlands is the prospect of an increase in nuisance biting flies and the potential for mosquito-borne disease transmission. In recent decades, England has been free of the transmission of infectious pathogens by mosquitoes, although it is less than 100 years since malaria was endemic in coastal marsh and fenland areas of England [15]. Furthermore, since the serious emergence of West Nile virus in North America in 1999 a great deal of attention has focussed on the role of both native and non-native mosquito species in the transmission of diseases to humans in Europe [16]. In the last five years continental Europe has witnessed its own local and ongoing transmission of West Nile virus to humans across large areas of the Eastern and Central Mediterranean. In 2012 there were 937 WNV cases in 16 countries in Europe and North Africa, with a further 785 WNV cases in 15 countries in 2013 [17]. European mosquitoes have been responsible for the transmission of *Plasmodium vivax* malaria in Greece [18], the emergence of African flaviviruses, such as Usutu virus in central Europe [19], and the prospect of European transmission of Rift Valley fever virus by European mosquitoes remains a grave concern of EU agriculture bodies [20]. In addition, two other arboviruses transmitted by European mosquito species occur cyclically in Europe: Sindbis virus in Scandinavia [21] and Tahyna virus in the Dyje and Danube river areas [22]; both of which are causative agents of human disease. Furthermore, the recent appearance of non-native invasive mosquitoes (e.g. *Aedes albopictus, Aedes aegypti, Aedes japonicus*) and their involvement in European transmission of dengue virus and chikungunya virus has raised the prospect of mosquitoes as a cause of public health concern in Europe [23,24].

One of the challenges, therefore, for wetland managers, those involved with flood alleviation and entomologists involved with public health assessment, is ensuring that existing and new wetlands, as well as flooding events (and flood risk plans), do not cause concern for public health disease risk either now or in the future [2]. Across Europe there are notable examples of how natural and unnatural flooding exacerbates mosquito nuisance and mosquito-borne disease issues. Such examples include increases in the abundance of *Aedes sticticus* and *Aedes vexans* following flooding events in the valleys of the main European rivers, such as the Dyje, the Danube and the Rhine [22,25], as well as enormous mosquito densities associated with seasonal flooding in the Swedish wetlands [26,27]. Massive increases in the abundance of *Aedes caspius* are also reported each year in the Camargue wetland system of France [28] and in the rice fields of the Piedmont Region in Italy [7].

UK initiatives to adapt to climate change include a number of field-based investigations of the impacts of flood alleviation, wetland creation and wetland management in England [2,5,29] and also in the Netherlands. The UK currently has 34 recorded species of mosquito, some of which have only been identified as new to the country in recent years [30-33]. A full list of British species and their favoured habitat are detailed in Table 1. Unlike the invasive *Aedes* species establishing in Europe, our endemic mosquito fauna exploit a range of habitats including permanent, temporary and container habitats, fresh or saline waters, flooded grassland or woodland habitats, tree-holes and underground sites. Some species show habitat specificity, whereas others are generalists. Some species require permanent aquatic habitats for survival, whereas others rely on a period of drying and can be pioneer species in new aquatic habitats. The impact of wetland creation, expansion and management will, therefore, impact each species in different ways. Here we discuss the impacts of three wetland initiatives in England in relation to future mosquito nuisance or disease vector implications.

**Table 1:** **Summary of the impact of wetland creation and management on British mosquito species, with a summary of potential for mitigation and possible nuisance/vector concern**

**Species**	**Status**	**Aquatic habitats**	**Impact of wetland creation**	**Impact of wetland management and possible mitigation**	**Human nuisance biting or vector concern**
*Anopheles algeriensis* Theobald	+	Fen, only in two local areas	None	N/A	Not considered an important nuisance species or potential disease vector
*Anopheles atroparvus* van Thiel	++	Coastal, brackish	No current evidence that newly created coastal wetlands created under managed re-alignment will be colonised by this species.	N/A	Causes nuisance in some coastal areas, however is of less concern than *Ae. detritus*. Has the potential to be a malaria vector, although the risk of local transmission is considered very low.
***Anopheles claviger*** **Meigen**	**+++**	Primarily breeds in permanent water such as ditches and pools, and generally favours heavily vegetated breeding sites. It may also be found in transient water habitats.	New permanent wetlands with ditches and pools will provide new habitats for this ubiquitous species. This species will also be favoured where ditches are not regularly brinked or slubbed, or if they are left to dry out.	This species can be kept in check by maintaining healthy ditches with plenty of predator competition, regular brinking of ditches to keep vegetation levels down and allow sunlight through.	This species is already widespread and is not currently significantly associated with nuisance biting. Although new wetlands might create more habitat, there is no evidence to suggest that it will become problematic.
***Anopheles messeae*** **Falleroni** ***/Anopheles daciae*** **Nicolescu**	**+++**	Breeds primarily in open sunlit permanent freshwater pools and ditches, including in seasonally flooded grassland.	New permanent wetlands with ditches and pools will provide new habitats for this ubiquitous species. This species also colonises the margins of open water in seasonally flooded grasslands, presumably the result of re-colonisation.	Brinking of ditches is associated with higher abundances of immatures of this species.	There is no evidence that this species is a nuisance biter; few adults are caught in mammal-lured traps or human landing catches, despite the local abundance of immatures. Although capable of transmitting malaria, owing to limited human biting this species is unlikely to be a concern.
				Flooding wet grassland in late spring provides new habitats for this species.	
*Anopheles plumbeus* Stephens	++	Tree-holes	None	N/A	Potential malaria vector, although not previously considered to be a principal vector.
**Species**	**Status**	**Aquatic habitats**	**Impact of wetland creation**	**Impact of wetland management and possible mitigation**	**Nuisance or vector concern**
*Aedes vexans* Meigen	+	Rare; flooded grassland	Unknown, too rare	N/A	Potential vector of Rift Valley fever virus, however, this species is currently very rare in the UK.
***Aedes cinereus*** **Meigen** ***/Aedes geminus*** **Peus**	**+++**	Flooded fen/grassland	Exploits a range of groundwater-fed summer flooded habitats, such as fens and wet grassland. How quickly they colonise new flooded grasslands is not yet known as very few immatures have been found in recently constructed wet grassland in the fens despite high adult densities in traps and resting in grazing exclosures. It is expected that colonisation will take place.	High groundwater levels in the summer will dramatically enhance the density of this species where it occurs. This was proven in the Cambridgeshire study.	The distribution of this species is patchy; however, where it does occur it can be a human biting nuisance. There is limited information on dispersal ranges. Owing to its anthropophagy and ornithophagy it has been implicated as a possible bridge vector of a number of arboviruses in Europe. This species will benefit from expansion of reedbeds, flooded meadows and seasonal summer flooding in open habitats.
				It is possible that the timing of flooding could be planned so that the eggs are left high and dry. Winter flooding rather than spring flooding would be less favourable for this species, as immatures tend not to appear until April. Draining of flooded areas during spring would significantly reduce the survival of immatures.	
*Aedes geniculatus* Olivier	++	Tree-holes	None	N/A	Not considered an important nuisance species or potential disease vector
*Aedes leucomelas* Meigen	+	Rare, only in a few locations	N/A	N/A	Too rare currently to be of concern as a nuisance or vector species
***Aedes caspius*** **Pallas**	**+++**	Coastal habitat; also flooded fen/grassland	Historical records of this species are mainly coastal with a few around London. However data from the fens show that it can be very common in flooded fen and newly created wet grassland. Furthermore, it has been recorded to colonise newly created freshwater (or weakly saline) habitats in managed re-alignment sites in estuaries.	Managing this species inland will be largely related to controlling water levels. High groundwater levels in summer, supplemented by precipitation leading to pooling and puddling in fen and wet grassland will provide submergence of dormant eggs. Summer flooding should be avoided	Can be a nuisance species, and although not considered a primary vector of Rift Valley fever virus, it has been implicated as main vector in Egypt. Further work to establish the role of this mosquito in potential arbovirus transmission is recommended, particularly considering its potential for wet grassland colonisation.
***Aedes annulipes*** **Meigen** ***/Aedes cantans*** **Meigen**	**+++**	Wet woodland	Not all wetland creation schemes intend to create wet woodland, however, where this does occur, consideration needs to be given to the impact of these species.	High amounts of winter flooding, and the persistence of flooded woodland in spring can significantly increase the densities of these species. Woodland ditches that are regularly slubbed and re-graded are less suitable for these species. However, if they are allowed to dry out and pools form, these habitats can become suitable breeding sties.	These species are serious nuisance biters of humans, and unlike other species, they bite during the day as well as at dusk. Although they are known to disperse from their habitat to find a host, there is no information on dispersal ranges. The siting of new developments near wet woodland, or the creation of new wet woodland near dwellings run the risk of exposing people to high biting rates, especially during June-August. Both species have been implicated as potential arbovirus vectors based upon their host-feeding habits (human and bird blood), however, they are not classed as a primary vector of WNV or SINV.
*Aedes communis* De Geer	+	Rare, few old records	Unknown, too rare	N/A	Too rare currently to be of concern as a nuisance or vector species
***Aedes detritus*** **Haliday**	**+++**	Coastal, brackish; also freshwater	Likely to be the principal mosquito colonising newly created coastal habitats, particularly at the spring high tide mark in isolated pools and in saline borrow ditches capturing brackish water.	This species exploits saline waters left by spring high tides. These areas generally occur at the limits of existing salt-marshes, in pasture or grassland subjected to flooding at high tides, or in vegetated ditches allowed to flood during high tides. Any regular tidal flushing usually reduces the suitability of these habitats for this species. Where possible, management of spring tide waters (through closure of sluices) mitigate the negative impact of this species. However, where tides regularly leave isolated pools with no drainage then biocidal treatment is required.	This species is a persistent biting nuisance and responsible for several mosquito control programmes in the UK. Although it is not considered a principal potential vector, its human and bird biting makes it a candidate vector of arboviruses. However its nuisance value alone makes it worthy of consideration and control.
*Aedes dorsalis* Meigen	+	Rare, coastal	Unknown, too localised	N/A	Not considered to be either a nuisance or vector species. Not widely distributed.
*Aedes flavescens* Müller	++	Coastal, brackish	Coastal marshes, although the species is not common	N/A	May cause nuisance biting, but not widespread, and not considered as an important potential vector.
*Aedes punctor* Kirby	++	Acid pools, bogs	Wet woodland sites on acid soils appear to favour this species. Therefore, not all wet woodland would be suitable, however in certain parts of England this species might benefit.	Winter/spring flooding of acid habitats, particularly in bog/mire/lowland moor areas can dramatically increase the numbers of this species. These habitats are naturally flooded by rainfall rather than groundwater, so management might be difficult. Dwellings close to such habitats will likely be impacted by wet winters/springs.	This species is not considered a principal vector of arboviruses although it is a nuisance species in areas adjacent to its favoured breeding habitat.
***Aedes rusticus*** **Rossi**	**+++**	Wet woodland, flooded rush pasture	This species would benefit from wet woodland creation and has also been found in new wet grassland habitats, particularly those dominated by rushes.	Spring flooding of wet grassland could provide a habitat for this species. Although they have not been found in high numbers, spring and winter flooding of wet woodland would create suitable habitat for this species.	This species is not routinely considered a potential vector, and although it does bite humans, its pest status is not as high as *Ae. cantans/annulipes* or *Ae. detritus*. However it will cause nuisance biting and will benefit from transient habitats subjected to winter/spring flooding.
*Aedes sticticus* Meigen	+	Wet woodland, very rare	Unknown, too rare. It may be unlikely that such a rare species would increase dramatically with wetland creation.	Timing of winter and spring flooding of wet woodland where this species occurs would be a consideration.	Nuisance and vector species elsewhere in Europe, but very rare in the UK. Where this species does occur, however, it can be a serious pest.
***Coquillettidia richiardii*** **(Ficalbi)**	**+++**	Permanent: ditches, vegetated pools	Newly created ditches with emergent vegetation will provide a suitable habitat for this species in time.	Owing to its enigmatic life cycle, the impact of management is difficult to determine. Vegetated ditches and ponds will provide a suitable habitat, but there is no clear evidence that management would be required, although this species can be abundant in July and cause a nuisance.	Can be a persistent biter after dark in high summer and is known to enter dwellings to bite. Not considered a principal arbovirus vector, but does feed on both birds and humans.
*Culex modestus* Ficalbi	++	Localised in coastal ditches	Not routinely found in newly created wetlands, although there are a few records from wet grassland.	Currently considered localised to North Kent and parts of Essex along the Thames estuary. Newly created ditches in this area would provide suitable habitat. Management of this species in permanent ditches might require biocidal control as there are currently no clear examples of the impact of water or vegetation management. However, emergent and floating vegetation appears a pre-requisite.	Known to be a nuisance species where it occurs along the Thames estuary and is also considered to be a principal vector of West Nile virus elsewhere in Europe.
*Culex territans* Walker	+	Rare, permanent habitats	Unknown, too under-recorded	N/A	Not considered an important nuisance species or potential disease vector
***Culex pipiens s.l.*** **Linnaeus** ***/Culex torrentium*** **Martini**	**+++**	All transient (e.g. dried ditches, flooded grasslands) and container habitats	The typical biotype of *Cx. pipiens* and *Cx. torrentium* colonise transient habitats post-flooding, and will, therefore, benefit hugely from wetland creation, particularly in the early pioneer stages of wetland development. It is unclear which species dominates and further studies are required. Any nutrient rich wet grassland or nutrient rich permanent habitat (i.e. polluted ditches, post-drought ditches, or sewage treatment reedbeds) will provide aquatic habitats for this species. In aquatic habitats hostile to predators/competitors these mosquitoes will increase to large densities. It is unclear whether the *molestus* form of *pipiens* will be affected by wetland creation. Container habitats for these species are unlikely to be affected by wetland creation.	For transient aquatic habitats, water-level management and precipitation will be crucial. Drying and re-wetting cycles of transient habitats, or unnatural drying of permanent habitats needs to be considered in relation to the rapid colonisation by these species. Raising water levels in wet grassland or wet fen in summer could be avoided to mitigate the impact of this species. Furthermore, permanent aquatic habitats should not be allowed to dry out. Mosquitoes associated with sewage treatment reedbeds may require biocidal control if deemed necessary, although this may not be efficient in nutrient-rich waters.	Neither the typical biotype of *Cx. pipiens* nor *Cx. torrentium* are nuisance species as they almost exclusively feed on birds. They are both considered important enzootic vectors of WNV and Sindbis virus respectively. In the event of such an outbreak, management of their populations will be crucial in managing the enzootic transmission of the viruses. The *molestus* form is also a potential WNV vector, but is unlikely to be affected by wetland management given its predilection for underground and cloistered container habitats.
*Culiseta longiareolata* (Macquart)	+	Rare, too few records	Unknown, too rare	N/A	N/A
*Culiseta fumipennis* (Stephens)	+	Rare, too few records	Unknown, too rare	N/A	Not considered an important nuisance species or potential disease vector
*Culiseta litorea* (Shute)	+	Coastal, rare, too few records	Unknown, too rare	N/A	Not considered an important nuisance species or potential disease vector
*Culiseta morsitans* (Theobald)	++	Permanent waters	It is expected that this species would benefit from the development of permanent waters, although they have been caught in such low numbers, there is insufficient data to determine the full impact of wetland creation.	There is little available information on the impact of wetland management, although vegetated ditches and reedbed that are subjected to drying and remain wet thereafter do provide a habitat for this species. It may not be necessary to control this species, but if required, certainly water level management will be crucial.	Not considered an important nuisance species owing to its largely ornithophagic tendencies. However, it is a potential enzootic disease vector in Europe and they have been reported to bite humans. However, they are heavily under-recorded in adult mosquito sampling.
*Culiseta alaskaensis* (Ludlow)	+	Northern species: rare, too few records	Unknown, too rare	N/A	Not considered an important nuisance species or potential disease vector
***Culiseta annulata*** **(Schrank)**	**+++**	Exploits a range of permanent, transient and container habitats	Will benefit from a range of wetland creation schemes, such as ditches subjected to drying, wet woodland with water persisting through to late summer, nutrient-rich wet grassland in late summer and drying nutrient rich reedbeds.	Wet woodland that remains wet throughout the year will provide a suitable breeding habitat for this species, which would also dominate in nutrient rich wet grassland. If this species is a problem then late summer flooding will be significant. Ditches allowed to dry and re-wet will also provide a suitable habitat. This species will also colonise polluted container habitats in urban areas, where they may be more of an issue.	This species is one of the most common nuisance species in the UK, although not necessarily biting in as high numbers as other species. It is large and owing to its colouration is often confused with some much smaller invasive species. Although not a principal arbovirus vector, its ability to feed on humans and birds (in urban areas) makes it a candidate vector.
*Culiseta subochrea* (Edwards)	+	Similar to *Cs. annulata*, likely under-recorded. Ecology not considered distinct from *Cs. annulata*	N/A	N/A	Not considered an important nuisance species or potential disease vector
*Orthopodomyia pulcripalpis* Rondani	+	Tree holes, rare	N/A	N/A	Owing to its ornithophagic tendencies, this species is not considered an important nuisance species or a potential disease vector

Key references: [5,29,32,34-46].

Status: +++ widespread, ++ more localised but locally abundant, + very focal or rare.

### Coastal wetland creation – sea level rise management

The winter of 2014 in southern England highlighted the risks posed to the British coastline by storm surges and high sea levels. In north-west Europe the prospect of sea-level rise, as well as the repeated incidence of coastal and estuarine flooding has led to the development of strategies to mitigate coastal flooding through the provision of new coastal habitat. This has prompted the implementation of managed realignment (MRA) schemes along the coast, which essentially involve replacing artificial hard coastal defences with natural soft defences, such as coastal habitats [8]. MRA is the deliberate process of altering flood defences to allow flooding of a presently defended area. This practice is now considered to offer long-term sustainable management of coasts and estuaries for a variety of stakeholders. It can reduce the loss of intertidal habitat due to coastal squeeze (fixed defences at the high-water mark reduce the extent of intertidal areas) and offer potential new habitat creation and re-creation opportunities [8]. It is also being promoted to mitigate loss of European protected coastal habitats, with MRA being directly implemented as compensation sites for direct loss, usually through the Environmental Impact Assessment (EIA) process and/or as part of an Appropriate Assessment under EU legislation [10].

An MRA scheme may take many forms, including a) retreating to higher ground, i.e., where a line of defence is breached/removed, allowing inundation up to the higher ground, producing a new intertidal area, b) constructing a set-back line of defence, thus protecting property landward of the defence, or land at lower altitude to the defence, c) shortening the overall defence length to be maintained, thus reducing costs or d) inundation through sections of defence whereby the breach allows inundation of land behind it through a defined gap, or through pipes with tidal flaps to allow an intertidal area to develop behind the defence.

The flood management and environmental benefits of MRA are manifold [8]. These include a) reducing flood risk or reducing the long-term costs of flood defence by minimising costs of repairing defences, b) ensuring long-term sustainability of defences by increasing natural flood and storm-buffering capability, c) reducing the costs of maintaining defences, particularly where existing defences are not economically viable, d) assisting with managing the effects of sea level rise by reducing the height of sea/estuary levels, and e) mitigating the effects of previous reclamations and of climate change (i.e., sea level rise), and thus offsetting the impact of coastal squeeze. Most significantly, MRA lead to creation of intertidal habitat, usually mudflat and salt marsh areas with the associated benefits for wildlife and protected species, as well as improving recreational value.

Coastal aquatic habitats have long been known to provide suitable habitats for brackish-water mosquitoes and historically, coastal marshes were considered to support anopheline mosquito populations that were responsible for local malaria transmission [15]. Studies in England surveyed the eight largest managed realignment sites (in Essex and the Humber) for mosquito habitats [5]. The apparent absence of anopheline mosquitoes exploiting aquatic habitats at all of these sites suggests that the risk of malaria associated with MRA sites is currently negligible. However, three of the eight sites support populations of two nuisance and potential arboviral vector species, *Aedes detritus* and *Aedes caspius*. These new breeding sites resulted from specific design aspects of the new sea wall; (a) an additional bank of ballast to mitigate wave action provided a linear habitat that supported significant numbers of mosquitoes, (b) during construction of the sea wall, saline borrow ditches were excavated and these now fill with brackish waters at spring high tides, and tidal waters collect on vegetated banks in culverts with minimal tidal flushing, and (c) isolated pools have been created through silt accretion or expansion of flooded zones to neighbouring pasture. Specific issues appear to be related to sites on a case by case basis, but in each case aquatic habitat that supports nuisance biting mosquito species has been created that, in some cases, reduced tourist visits and/or impacted on the local community.

There is likely to be some nuisance biting associated with sites that support flooded habitats that are not regularly flushed by the tide, and a management plan may be needed to deal with these sites if they continue to promote nuisance biting by *Ae. detritus* and *Ae. caspius*. Similar controls would also be needed if, in the future, these species become implicated in disease transmission. Ideally, consideration should be given to the creation of potential aquatic mosquito habitats prior to any MRA construction, as part of the Environmental Impact Assessment, with further consideration as to how such habitats could be minimised. Ongoing monitoring of such sites for mosquitoes should be instituted as part of regular faunal and floral monitoring and public health risk assessment for each site. It is possible that using field generated evidence-based findings, targeted mosquito management for specific nuisance species could be put in place that involved small changes to flooding regimes or sluice management. For example at one site in Essex, closing of sluices to prevent over-flooding of ditches at spring high tides would minimise vegetation flooding and thus mitigate the problems encountered with *Ae. detritus*. However, the management of each site must be considered on a case-by-case basis.

### Urban wetland creation – mitigation for urban development

The drivers for developing new wetlands in urban areas are manifold [11]; e.g., ecological mitigation to offset loss of wetland habitat for European protected species, local urban flood-risk management, particularly where housing developments are constructed in areas prone to flooding, and improvement in local public access to natural areas has led to the creation of extended urban green and blue space. In some cases there is a drive to provide sustainable urban drainage, which may involve harnessing the ability of wetlands to sanitise sewage to minimise local environmental pollution. Urban green and blue space is increasingly being promoted to mitigate the urban heat island effect.

There is a dearth, however, of field-based evidence on the impact of urban wetland creation schemes on mosquito populations in Europe. Studies in England have begun to address this [34]. New wetlands created in urban and peri-urban locations in England generally take a similar form, usually directed by the requirements of protected wildlife species and government legislation. In the UK, permanent water bodies in the form of ponds or ditches are created for Great Crested Newts and Water Voles. Reedbeds and open water areas are created for migrant warblers and wildfowl. Transient winter-flooded scrapes are created for wading birds such as Lapwing. In addition, many sites also have a constructed reed-bed, separated hydrologically from the rest of the wetland, which is used to treat sewage from the local community, particularly during flash-flood events to reduce environmental contamination.

Studies on the impact of the creation of such habitats on local mosquito populations in England are revealing. These aquatic habitats are hugely responsive to changes in the water table caused by precipitation. Colonisation by mosquitoes of permanent habitats (such as open water, marginal reed areas and ditches) appears to be extremely slow, particularly during the first two years before marginal, floating and submerged vegetation has developed, when these sites can become suitable for anophelines. Furthermore, fluctuations in water depths of these groundwater-fed permanent habitats can, during the early phases of reed planting, lead to drying out and/or flooding of planted emergent vegetation, such as reeds, which further delays mosquito colonisation. In contrast however, transient aquatic habitats (subjected to repeated wetting and drying), particularly those with a vegetated substrate, are quickly exploited by mosquitoes, and densities of mosquitoes are further enhanced by periods of drying and re-wetting that exclude all competitors and predators. In the UK, the dominant mosquito species in this ephemeral habitat are *Culex pipiens* s.1./ *Culex torrentium*– which are known elsewhere in Europe for acting as an important enzootic (transmission between birds) vector of West Nile virus and Sindbis virus. Densities of immature *Culex* were reported to be 33 times higher in transient versus permanent habitats, and 41 times higher in vegetated compared with unvegetated habitats [34].

However, the situation is slightly different in reedbeds created for sewage treatment. These are designed to remove phosphorus from sewage effluent, with the resultant water entering the river system. These tend to be self-contained systems, separate from the rest of the urban wetland. Crucially they include an unvegetated stilling basin that holds the sewage prior to passage through the reedbed. Studies in England found that these treatment reedbeds contributed significantly to the local density of mosquitoes. This was particularly the case following an influx of sewage in the stilling basin. The incoming sewage acts as a productive food source for *Culex pipiens,* with immature mosquito densities reaching several thousand per litre of surface water. Overall, mean densities of immature *Culex* were reported to be 80 times higher in the stilling basin compared to all other aquatic habitats created in the new urban wetland, with larval densities 150 times higher in the sewage filled stilling basin compared to the rest of the sewage treatment reedbed [34].

In the event of an outbreak of West Nile virus the numbers of mosquitoes exploiting these stilling basins needs to be considered. Several months following a sewage input event, the high nutrient levels, coupled with high summer temperatures, can create blooms of blue-green algae, which also supports immature mosquitoes, but to a much lesser degree [34]. Throughout the rest of the vegetated sewage treatment reedbed mosquito numbers tend to be low (including *Anopheles claviger, An. maculipennis* s.l., *Culex pipiens* and *Culiseta annulata*), as there is an assemblage of predators. However, such habitats should not be allowed to dry out as this would negatively impact populations of natural predators.

Currently no consideration is given to the impact of urban wetland creation on mosquito numbers or future vector-borne disease risk in the UK, and yet this should be a primary consideration of all future wetland construction applications and included in the environmental impact assessment. Groundwater management and effluent inflow will contribute to the majority of culicine populations and appropriate targeted management using biocidal control should be used to manage these species.

### Freshwater wetland management and expansion including arable reversion

The Wetland Vision for England [9] outlines exciting plans to restore existing wetlands and create new wetlands from areas currently under agriculture. This is an attempt to provide an increased resource for biodiversity, assist with alleviating inland flooding and re-connect extant nature reserves to ensure that wildlife species are able to adapt to the impact of climate change. There has long been a recognition that extant highly-biodiverse wetland habitats require ongoing management to maintain a range of wetland communities with various seral stages, including tall fen, fen meadow, rush pasture, swamp, open water, permanent and temporary ditches, and wet woodland. An unmanaged wetland eventually becomes woodland. These different wetland types support a varied range of mosquito species. The impact of seasonal rainfall and/or management of water levels can impact significantly on the survival and abundance of these species. Often the impact varies between sites, so that an assessment of wetland management and extreme weather events on mosquito numbers is only possible on a case-by-case basis, although there are some general principles that will now be discussed.

In addition to management of extant wetlands, there is also recognition of how these habitats have become fragmented across a landscape through the expansion of agriculture. One of the goals, therefore, for climate change adaptation is to de-fragment these wetlands to ensure that wildlife species can adapt to changes in climate and have many more staging posts to ensure their survival. One of the key components of Wetland Vision projects, such as the Great Fen project and the Wicken Fen vision (both in Cambridgeshire, UK) is to expand existing wetlands through arable reversion. This involves purchasing neighbouring arable land (often land that had previously existed as wetland prior to drainage) and re-seed and re-wet it; often managing it as wet grassland with grazing animals (an important food source for mosquitoes).

It is important to consider the variation in general life-histories of mosquito species and how these relate to wetland types [2,29,35,36]. Some mosquito species exploit only permanent wetlands such as ponds and ditches. Larger lakes tend to be inimical for mosquitoes as they are subject to surface movements that prevent immature mosquito survival. Furthermore, certain wetland types (such as reedbeds) with extensive drawdown zones (area at the edge of a body of water that is frequently exposed to the air due to changes in water levels) do not tend to support mosquitoes. A vegetated substrate or the presence of floating or emergent vegetation is generally required to support mosquitoes in permanent wetlands. Another group of mosquito species thrive in temporary water that is subjected to seasonal flooding and drying. Mosquitoes of wet woodland tend to exploit winter flooded habitats, with immature development occurring during late winter and early spring prior to summer drying. Mosquitoes of wet grassland remain dormant during winter as eggs, awaiting summer floods, upon which immatures develop in late spring for a summer emergence of adults. Wetlands that routinely dry and re-wet tend to have the associated groups of invertebrates adapted to this habitat which are also competitors and predators of mosquitoes, however, the erratic nature of such ephemeral habitats leads to higher than average mosquito densities. For healthy permanent wetlands, however, mosquito numbers are maintained by the food web, owing to the multitude of mosquito predator species. One of the main challenges, however, is associated with extreme events, such as drought, which results in the relatively rare drying of permanent wetlands, followed by a re-wetting event and a subsequent dramatic increase in mosquito numbers in the absence of competitors and predators [3].

If we now relate this variability in habitat conditions that are suitable for mosquito breeding, to wetland management we can see that maintaining permanent wetlands throughout the year is crucial to minimise the effect of permanent wetlands as a source of nuisance mosquitoes. Studies of the Great Fen project [29] have shown that permanent wetlands maintained low densities of anopheline mosquitoes (e.g. *An. maculipennis* s.l., *An claviger*). However, if ditches dried during a drought event, upon re-wetting, large numbers of culicines colonised (e.g. *Cx. pipiens, Cs. annulata*) the area. Anophelines accounted for 98% of all mosquitoes collected from the ditches that remained wet, however, ditches subjected to drying were dominated by culicines (93%). The process of slubbing (digging out ditches) can prevent this and should be promoted. The vegetation surrounding ditches is also managed (a process known as brinking), and seasonal brinking can affect the densities of mosquito immatures by raising water temperatures, which favours some species (particularly *An. maculipennis* s.l.). Significantly more immature anophelines were shown to occur in ditches that had been subjected to cutting of marginal vegetation in late spring [29].

With respect to temporary water bodies, the timing and intensity of flooding (through precipitation or raising water levels) can affect a range of mosquito species. Prolonged winter and spring flooding in wet woodland habitats can prolong the period in which immature mosquitoes can complete development and thus increase the density of nuisance woodland mosquitoes.

Summer precipitation favours high density populations of floodwater mosquito species, particularly in wet grassland habitats where the effects can be profound due to there being two phases of development in these species [29]. Firstly the mosquitoes that deposited eggs the previous year in areas prone to flooding emerge first, usually in synchrony. This can lead to large numbers of nuisance and vector species (e.g. *Ae. caspius, Ae. vexans, Ae. sticticus*). This is usually followed by colonisation of a different set of species (e.g. *Cx. pipiens, Cs. annulata*) that oviposit on open water and thrive in drying nutrient rich waters provided there is a vegetated substrate or marginal vegetation. Late spring rainfall appears to be crucial in determining the retention of water levels in these habitats. An already high water table, exacerbated by summer rains, can be ideal for high densities of floodwater mosquitoes. A flooded river in a river valley has a similar effect.

Late summer rains can also be retained by wetland managers in wet grassland habitats through a series of sluices, thus retaining water on the grasslands through winter to promote winter visiting wildfowl. The timing of the commencement of this flooding is crucial. Flooding as late as September and October can still promote immature mosquito development, particularly if unseasonably high temperatures promote rapid development through to emergence of nuisance adults [29]. These are important considerations during arable reversion to wet grassland. A very wet late spring and summer, or a wetland subjected to management of summer water levels will have a profound effect in supporting nuisance and vector mosquitoes. Wetlands that either stay very wet, or remain very dry will likely have a lower impact. There appears to be a depth threshold in these wet grasslands above which mosquitoes cease to exist [29]. Therefore, it may be possible that during an outbreak situation, raising water levels above specific depth thresholds will make the habitat inimical for mosquito development. However, natural or unnatural re-wetting of wet grassland during summer will contribute to large numbers of nuisance and potential vector species. These findings have important implications for understanding the impact of extreme weather events such as drought and flooding. However, it is important to consider that through wetland management, aquatic habitats are subjected to flooding and drought on a regular basis, often independent of weather conditions.

In summary, different types of wetland support a range of different mosquito species, depending upon the range of wetland types and the size and age of the wetland. Management of water levels at specific times of year can have a profound effect on mosquito densities. Understanding the impact of seasonal rains and water level management needs to be considered on a case-by-case basis using the generic principles outlined above. Wetlands close to dwellings may even need a mosquito management plan. This will be increasingly important during disease outbreak situations.

## Review and conclusion

It is vital that wetland creation, expansion and management plans take into account the effects that wetland management might have on mosquito populations, nuisance-biting levels, and public and veterinary health. It is also necessary that such biodiversity initiatives have the knowledge and tools to enable them to assess and manage this impact as their work proceeds. It is crucial that environmentally-friendly mitigation strategies and wetland site locations are chosen with mosquito life histories in mind in order to minimise or avoid potentially deleterious effects. The environment sector recognises that there is a need for an evidence-base to inform future wetland creation and management initiatives and public health entomologists in the UK are now working to develop this evidence base and appropriate guidance with wetland experts. Applied correctly and, where possible, exploiting biodiversity and wetland management could become key tools in keeping mosquito populations at desirable levels. However, this can only be achieved if environmental impact assessments for new wetlands consider mosquitoes in their health chapter, and that wetland management plans develop a contingency for mosquito management (through wetland management strategies rather than only biocide usage) in the event of an outbreak. There will be no time to devise such plans in the face of an outbreak, so prior planning is highly recommended. The fact that wetlands do produce aquatic habitats for mosquitoes should be accepted, but it should also be recognised that evidenced-based management strategies targeted at key mosquito species will be crucial in managing disease outbreaks (or nuisance mosquito problems). Furthermore, a combined effort from environmental and public/veterinary health sectors is required to ensure that in the event of a mosquito-borne disease outbreak biocidal and environmental control of mosquitoes is appropriate and targeted to ensure maximum efficacy at minimal cost. A summary of the possible impact of wetland creation, wetland management and possible mitigation and perceived nuisance or vector concerns are given for all UK species in Table 1. It should be noted that much more research is required to refine these recommendations and to understand further the impact of wetlands on mosquitoes. There is also a requirement to better understand the biological and ecological complexities of mosquito species complexes through better molecular identification. In conclusion, developing this evidence-base ahead of a disease outbreak is currently a primary objective of public health and environmental agencies.
